# Disruption of Exocytosis in Sympathoadrenal Chromaffin Cells from Mouse Models of Neurodegenerative Diseases

**DOI:** 10.3390/ijms21061946

**Published:** 2020-03-12

**Authors:** Antonio M. G. de Diego, Diana Ortega-Cruz, Antonio G. García

**Affiliations:** 1 Instituto Teófilo Hernando, Departamento. de Farmacología, Facultad de Medicina, Universidad Autónoma de Madrid, 28029 Madrid, Spain; antoniomiguel.garcia@uam.es (A.M.G.d.D.); diana.ortega@ifth.es (D.O.-C.); 2 Instituto de Investigación Sanitaria, Hospital Universitario de La Princesa, 28006 Madrid, Spain

**Keywords:** chromaffin cell, neurodegenerative diseases, sympathoadrenal axis, exocytosis

## Abstract

Synaptic disruption and altered neurotransmitter release occurs in the brains of patients and in murine models of neurodegenerative diseases (NDDs). During the last few years, evidence has accumulated suggesting that the sympathoadrenal axis is also affected as disease progresses. Here, we review a few studies done in adrenal medullary chromaffin cells (CCs), that are considered as the amplifying arm of the sympathetic nervous system; the sudden fast exocytotic release of their catecholamines—stored in noradrenergic and adrenergic cells—plays a fundamental role in the stress fight-or-flight response. Bulk exocytosis and the fine kinetics of single-vesicle exocytotic events have been studied in mouse models carrying a mutation linked to NDDs. For instance, in R6/1 mouse models of Huntington’s disease (HD), mutated huntingtin is overexpressed in CCs; this causes decreased quantal secretion, smaller quantal size and faster kinetics of the exocytotic fusion pore, pore expansion, and closure. This was accompanied by decreased sodium current, decreased acetylcholine-evoked action potentials, and attenuated [Ca^2+^]c transients with faster Ca^2+^ clearance. In the SOD1^G93A^ mouse model of amyotrophic lateral sclerosis (ALS), CCs exhibited secretory single-vesicle spikes with a slower release rate but higher exocytosis. Finally, in the APP/PS1 mouse model of Alzheimer’s disease (AD), the stabilization, expansion, and closure of the fusion pore was faster, but the secretion was attenuated. Additionally, α-synuclein that is associated with Parkinson’s disease (PD) decreases exocytosis and promotes fusion pore dilation in adrenal CCs. Furthermore, Huntington-associated protein 1 (HAP1) interacts with the huntingtin that, when mutated, causes Huntington’s disease (HD); HAP1 reduces full fusion exocytosis by affecting vesicle docking and controlling fusion pore stabilization. The alterations described here are consistent with the hypothesis that central alterations undergone in various NDDs are also manifested at the peripheral sympathoadrenal axis to impair the stress fight-or-flight response in patients suffering from those diseases. Such alterations may occur: (i) primarily by the expression of mutated disease proteins in CCs; (ii) secondarily to stress adaptation imposed by disease progression and the limitations of patient autonomy.

## 1. Introduction

Synaptic disruption and altered neurotransmitter release are common pathogenic features in neurodegenerative diseases (NDDs) such as Huntington’s disease (HD) [1], amyotrophic lateral sclerosis (ALS) [2], and in Alzheimer’s disease (AD) [3]. For instance, in Alzheimer’s disease (AD), both pre- and postsynaptic alterations and synaptic loss are major correlates of disease severity [4]. Additionally, presenilins play a critical role in the regulation of neurotransmitter release [5]. Furthermore, it seems that amyloid beta (Aβ) also plays a role in the regulation of synaptic function [6,7]. In the light of these studies, it seems plausible that alterations of these proteins may produce synaptic alterations that underlie AD pathogenesis; in fact, AD has been pathogenically considered as a synaptic failure [8].

In amyotrophic lateral sclerosis (ALS), augmented glutamate release and glutamate receptors have been implicated in motoneuron death [9]. In this sense, it is worth noting that in the R6/2 mouse model of HD, brain dopamine release is severely compromised [10]. Also, altered striatal amino acid neurotransmitter release has been reported in R6/1 mice [11].

As the activity of the peripheral sympathoadrenal axis is exquisitely controlled at specific sites in the brain cortex and hypothalamus, alterations of the exocytotic release of neurotransmitters may also occur at peripheral sympathetic neurons and adrenal medullary chromaffin cells. As the hypothesis raised here implies the alteration of the sympathoadrenal axis, in this review we will briefly comment on its connections with the central nervous system (CNS). Then, we will review the alterations undergone at the bulk secretion of catecholamines and the fine kinetics of single-vesicle exocytotic events in three transgenic mouse models of NDDs, and end with a comment on the altered exocytotic events in mouse CCs overexpressing some pathological proteins linked to NDDs. We will finally formulate a hypothesis on the potential impact of these alterations on the control of the stress fight-or-flight response by the sympathoadrenal axis in patients suffering HD, ALS, or AD.

## 2. Central and Peripheral Control of the Stress Fight-or-Flight Response

The body homeostasis, the flight-or-fight response, and the functional unitary nature of the sympathoadrenal system are three concepts introduced by Cannon [12]. The physiological control of these functions is exerted by the autonomic nervous system through its two divisions: parasympathetic and sympathetic. The axons of the sympathetic neurons that form the prevertebral and paravertebral ganglia [13] innervate and regulate most organs and blood vessels through the release of noradrenaline. The adrenal medulla is the amplifying arm of the sympathetic nervous system; it is formed by a collection of chromaffin cells (CCs) that, in their chromaffin vesicles, store the catecholamines noradrenaline and adrenaline, that are released into the blood stream upon stimulation by acetylcholine (ACh), being released by splanchnic nerve terminals that innervate the noradrenergic and adrenergic CCs. 

Of interest is the well-established concept of the presence in the adrenal medulla of separate populations of noradrenergic and adrenergic cells [14,15,16]. Elegant experiments using double-virus transneuronal labelling showed that the secretory activity of CCs is regulated by neuron collections located at the brainstem and the hypothalamus [17]. Furthermore, the burst pattern stimulation of the trigeminal nucleus caudalis preferentially released adrenaline [18]. Additionally, the selective adrenaline release was shown to be also regulated by the autonomic areas located in the medulla oblongata [19,20], the hypothalamus [21,22], and the cerebral cortex [23]. On the other hand, the stimulation of other regions of the medulla oblongata [20] or the hypothalamus [21,22] selectively regulates the release of noradrenaline. Furthermore, the more cephalic preganglionic outputs from the spinal cord innervate adrenergic CCs and the more caudal do innervate noradrenergic CCs [24,25]. These studies support the view that the differential secretion of noradrenaline or adrenaline is regulated by the different input patterns that each CC subtype receives from specific brain regions during stress. 

The fight-or-flight response is quickly activated at cortical and hypothalamic brain sites during an alarming acute stressful conflict, namely intense fear, exercise, or struggle. Through the activation of practically all organs of the body, via their adrenergic alpha and beta receptors, the noradrenaline released from sympathetic nerve terminals and adrenal chromaffin cells, and the adrenaline released from chromaffin cells into the circulation, trigger the fight-or-flight response to fight or run away from danger; pupils dilate to increase visual acuity, and heart rate, myocardial contraction, and blood pressure increase to switch blood to skeletal muscles to insure maximal performance and motor responsiveness. This is achieved by producing vasodilation in the skeletal muscles and vasoconstriction in the skin and visceral vasculature. At the same time, bronchodilation is produced to augment the oxygen supply and glucose is mobilised from the muscle and liver to increase glycemia; thus, metabolic activity is increased in practically all cells of the organism, to ensure a coordinated response for survival.

## 3. Bulk Release of Catecholamines from Chromaffin Cells of Mouse Models of NDDs

**Figure 1:**
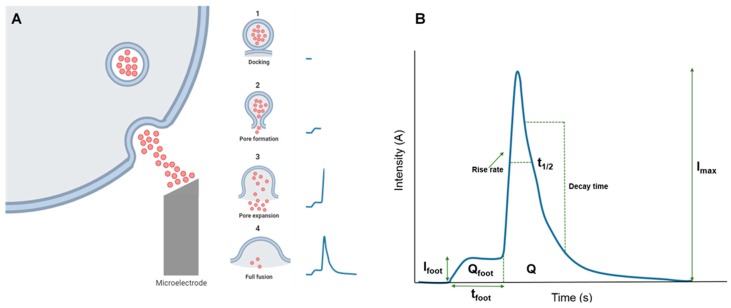
Detection of single-vesicle catecholamine release with a carbon-fiber microelectrode (panel **A**). In this technique, the process of exocytosis is recorded by placing a polarized microelectrode close to the cell membrane, which oxidizes the released catecholamines. At the right of the cell scheme, the sequential time course of the generation of the exocytotic event is shown: 1, vesicle docking to the plasma membrane; 2, pore formation, with a small release of catecholamine causing a mild baseline elevation, the so-called spike-foot; 3, pore expansion and full secretion of vesicle contents (fast rise of the spike); 4, pore closure and spike relaxation to baseline. The different kinetic parameters of the spike are shown in panel (**B**). In several neurodegenerative diseases (NDDs), mouse models spike kinetic changes in a characteristic manner. (Adapted from de Diego and García, 2018, [27]).

The fine analysis of single-cell exocytosis was achieved first at the laboratory of R. M. Whightman. This was achieved by placing a carbon-fiber microelectrode placed adjacent to a CC (Figure 1A), and the amperometric detection of catecholamines through their electro-oxidation. The authors concluded that the individual spikes represented the quantal secretion of catecholamines from single storage vesicles (Figure 1B) [26].

Pulses of 1 min with supramaximal 100 µM ACh (the physiological neurotransmitter at the splanchnic nerve-CC synapse) evoked exocytotic responses consisting of an initial fast burst of amperometric spikes, followed by sparser spikes distributed along the 1-min stimulation recording period. The overall responses can be expressed as a cumulative quantal release of catecholamines in two analytical approaches: first, secretory spike number (SN) counted at 5-s intervals along the 1-min traces, an indication of the number of vesicles available for secretion at the ready-release vesicle pool (RRP, [28]); and second, the summatory areas of spikes, also calculated at 5-s intervals, an indication of the total cumulative catecholamine release amperometrically monitored (Qamp in pC).

In CCs from 7 month-old (7 m) R6/1 mice, a model of HD with phenotypic signs of disease stages (i.e., decreased time to fall in the rotarod test), SN, as well as Qamp were notably decreased, reaching a plateau sooner than in the age-matched wildtype mice (WT) CCs. Fewer secretory spikes indicated either a lesser vesicle number and/or a deficient transport of secretory vesicles from the reserve pool (RP) to the RRP, in order to replenish it. It is also compatible with a smaller quantal size of individual vesicles. A reduced number of vesicles is compatible with decreased levels of dopamine-beta-hydroxylase (DBH, a marker of secretory vesicles) of 6 m R6/1 mice. A lesser Qamp is in line with a drastic decrease of adrenaline and noradrenaline in the adrenal glands, and with a halved Q in R6/1, with respect to WT mice. It seemed, therefore, that a slower time course of secretion with a drastically decreased cumulative secretion was due to both lesser vesicle number as well as smaller quantal size in the CCs of 7 m R6/1 mice, with respect to age-matched WT mice [29].

A somewhat distinct picture emerged from a study carried out in CCs from the SOD1^G93A^ mouse model of ALS, at phenotypic advanced disease stages (postnatal day 100, P100) [30]. A lesser number of spikes were counted in this study (1357 spikes from 69 SOD1^G93A^ CCs, versus 2034 spikes from WT CCs). Although a time-course analysis of SN was not done, the lesser total spike number suggested that the lesser vesicle number underwent exocytosis in the SOD1^G93A^ CCs, with respect to WT. Opposite the R6/1 CCs, the SOD1^G93A^ CCs exhibited a faster and higher time-course of cumulative secretion, that could be explained by the 52% increase of quantal size of individual vesicles. It seems therefore that the SOD1^G93A^ CCs at disease stages have exocytotic responses to ACh with a lesser number of vesicles but a higher cumulative secretion of catecholamines, due to a higher quantal size, with respect to WT CCs of matched age.

## 4. Kinetics of Exocytotic Fusion Pore, Pore Expansion, and Closure in CCs from Mouse Models of NDDs

An amperometrically monitored spike is frequently preceded by a small foot. These two phases result from the same fusion event: the foot arises from the slow release of catecholamines through a so-called fusion pore that occurs at an early step of exocytosis [31]. The fusion pore was postulated to be a gap-junction-like structure that first forms a nucleus for fusion and, after some delay, dilates to generate the fast full secretory spike (Figure 1B) [32,33]. 

In the HD R6/1 mice, the number of spikes preceded by a foot was similar to WT, 58%; however, I_foot_, T_foot_, and Q_foot_ were 25%, 26%, and 36% smaller respectively, meaning that the fusion pore stabilized quicker to undergo full expansion more rapidly in R6/1 cells, with respect to WT cells [29].

Multiple-spike events and the rate of flickering were similar in WT and the SOD1^G93A^ ALS CCs, around 4% and 7%, respectively. Spikes with foot were slightly higher in the latter, 66% versus 59.6%; I_foot_ was 23% lower, t_foot_ was 31% higher, and Q_foot_ was similar. This means that in the SOD1^G93A^ mouse, the fusion pore stabilized faster, with respect to the R6/1 HD mice [30].

In the APP/PS1 mouse model of AD, the frequency of spikes with foot was similar to the WT mice (around 63%). I_foot_ and T_foot_ were somehow smaller, but the real difference was found in Q_foot_, which was halved in APP/PS1 CCs (28 fC) with respect to WT cells (59 fC). This means a faster stabilization of the fusion pore with a quicker transition to pore expansion in APP/PS1 cells [34].

Stabilization and expansion of the exocytotic fusion pore gives rise to a full spike, an indication of the quantal release of the catecholamines per single secretory storage chromaffin vesicle [31]. The kinetic parameters monitored in each individual spike indicate deep changes between the WT and transgenic CCs. With respect to the WT cells, the mean rise rate of the R6/1 spike was 34% faster, with 35% faster decay, 28% smaller t_1/2_, 50% smaller Q, and 37% lower Imax. This means that the R6/1 spike was substantially smaller and faster than its WT counterpart, suggesting a much lower release of catecholamines per vesicle undergoing exocytosis [29].

The SOD1^G93A^ spike exhibited a 40% smaller rise rate and 17% lower Imax; however, the rest of the parameters were substantially higher: 61%, 55% and 52%, respectively, for decay time, t_1/2_, and Q, with respect to the WT. This suggests a smaller and wider spike, with a higher secretion of catecholamines per vesicle, but at a slower rate [30]. The opposite was true for APP/PS1 CCs that showed 40%, 40% and 55% decreases of decay, t_1/2_, and Q, respectively, meaning a faster but smaller amount of catecholamine release per single spike [34].

## 5. Changes in Ion Currents and [Ca2+]c Signaling

The fine-tuning of calcium (Ca^2+^) homeostasis in CCs is tightly controlled by a functional triad comprising the voltage-activated calcium channels (VACCs; Ca^2+^ entry into CCs), the endoplasmic reticulum (ER), and the mitochondria (Ca^2+^ redistribution into organelles) [35]. In some of the studies referred to above, whole-cell calcium currents (I_Ca_), sodium currents (I_Na_), and changes of cytosolic calcium concentrations ([Ca^2+^]_c_), have also been explored [35].

In SOD1^G93A^ CCs, augmented whole-cell calcium current (I_Ca_) and decreased sodium current (I_Na_), were observed. This correlated with higher [Ca^2+^]_c_ transients and decreased CC excitability [30]. We also found notable changes in the R6/1 CCs, namely a decreased I_Na_, unaltered I_Ca_, and faster [Ca^2+^]_c_ clearance [29].

These changes in ion current and [Ca^2+^]_c_ clearance are difficult to correlate with the changes observed either in bulk exocytosis or the kinetics of single exocytotic events. However, we can be certain that in mouse models of NDDs, alterations are not restricted to the secretory machinery and the last steps of exocytosis at the subplasmalemmal exocytotic sites. Rather, these alterations also occur in cell excitability and ion currents; these are particularly notable in I_Na_, which is severely depressed in both SOD1^G93A^ and R6/1 CCs.

## 6. Some Pathological Proteins Associated with NDDs Are Also Expressed in CCs to Modify Exocytosis

Some pathological proteins associated with NDDs are clearly expressed in CCs. This is the case for huntingtin in R6/1 CCs, which is abundantly expressed in the cytoplasm and nucleus. Of interest is the observation that huntingtin is expressed even at presymptomatic disease stages. This may explain that at these stages there was already a pronounced decrease of bulk secretion [1,29]. The mutated enzyme SOD1^G93A^ is also expressed in CCs of the ALS mice (unpublished, doctoral thesis I. Méndez). Contrarily, in CCs from the AD model APP/PS1 mice, Aβ pathology was not present in APP/PS1 mice, although Aβ aggregates were certainly found in the cerebral cortex [34].

Some other pathological proteins are naturally expressed in CCs or have been transfected and overexpressed to inquire about their role in regulating exocytosis. Thus, pathology associated with α-synuclein has been found in the adrenal medulla of Parkinson`s disease (PD) patients, as in CCs endogenously expressing the protein [36]. Also, α-synuclein overexpression in the mouse CCs promoted fusion pore dilation [37] at the time it decreased exocytosis [38].

On the other hand, huntingtin-associated protein 1 (HAP1), localizes in adrenal CC vesicles [39]. The fact that HAP1 deletion decreases exocytosis in mouse CCs prompted experiments showing a smaller pool of ready-release vesicles and a smaller fraction of docked vesicles [40].

From the experiments in transgenic mouse models of NDDs and the endogenously expressed or transfected proteins, it seems clear that some pathological proteins that are being associated with the pathogenesis of NDDs are also expressed in adrenal CCs to disrupt exocytosis and the physiological release of catecholamines.

## 7. A Hypothesis on The Implication of The Sympathoadrenal Axis in The Pathogenesis of NDDs

Communication between neurons, and among neurons and the cells they innervate to control their functioning, secures body homeostasis and the responses to external and internal stimuli in health and disease [41]. The structural and functional basis for such communication is an electrochemical language formed by action potentials, cell depolarization, Ca2+ entry, and the release of chemicals either locally at the synaptic gap (the neurotransmitters) or at a distance through the circulation (the catecholamines in the adrenal medulla and other hormones). Considering that the stimulus-secretion coupling process in adrenal CCs is controlled by the brain cortex and hypothalamus via sympathetic spinal cord outputs and the splanchnic nerves, it seems plausible that synaptic disruption and altered neurotransmitter release at central areas occurring in NDDs may also impinge peripheral neurosecretory exocytotic events in different parts of the sympathoadrenal axis.

Due to the ease of isolation and culture of adrenal CCs, and taking into account that they are endowed with the reach collection of ion channels and action potentials inherent to neurons [42], it is of no surprise that CCs have extensively been used to explore the tiny alterations of the pre-exocytotic and exocytotic mechanisms underlying the release of catecholamines. So, the alterations of bulk exocytosis, and the kinetics of single exocytotic events in CCs from mouse models carrying a mutation of patients suffering from the NDDs described here, as well as those alterations elicited by pathological proteins associated with those diseases, support the hypothesis that “adrenal CCs and therefore, the sympathoadrenal axis controlling their functions, sense and mimic the alterations undergone by different synapses in the CNS of patients with NDDs”. Such alterations may occur in the pathogenic context of the following pathways: (i) disruption of the collections of neurons that control centrally the activity of the sympatho-adrenal axis at the brain cortex and/or the hypothalamus; (ii) a kind of “propagation” of disease proteinopathies from central to peripheral cells; (iii) impaired stress responses imposed by the limitations of body functions inherent to the progression of NDD pathology; (iv) mutant proteins that are expressed by the CCs themselves; and (v) hypoxic conditions linked to microvascular pathology that may exert drastic changes in CC functions.

## 8. Conclusions and Perspectives

**Figure 2:**
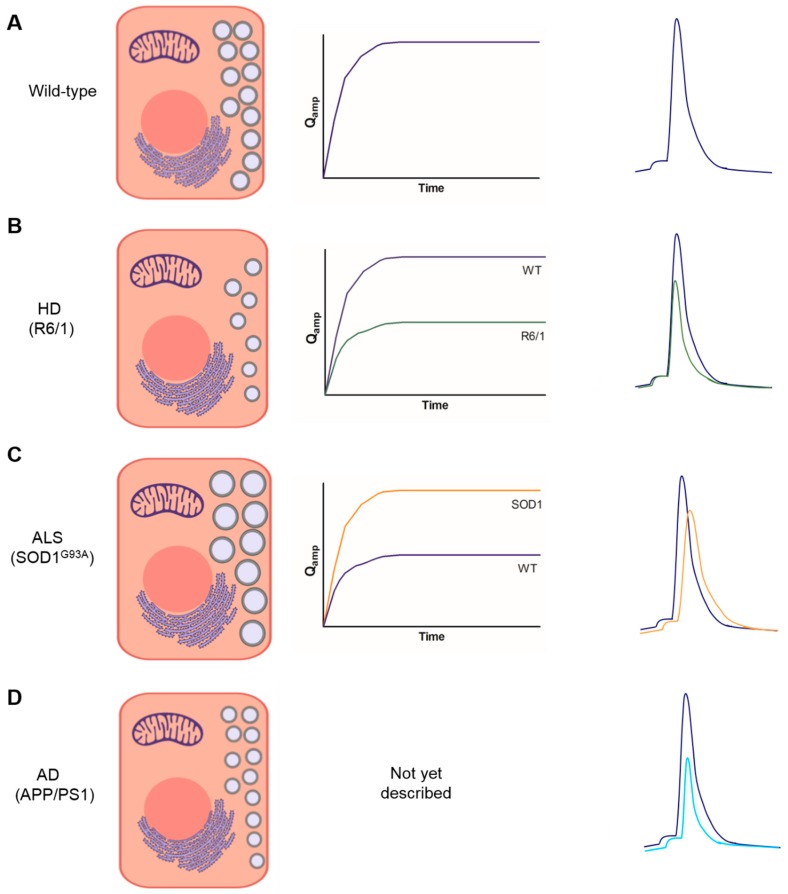
Schematic representation of altered exocytosis in chromaffin cells (CCs) from wildtype mice (WT) and mice carrying a mutation of Huntington’s disease (HD, R6/1), amyotrophic lateral sclerosis (ALS, SOD1^G93A^), and Alzheimer’s disease. CCs were stimulated with 1-min pulses of acetylcholine (ACh); bulk secretion (summatory areas of all spikes recorded in 1-min ACh stimulation) is represented in the middle traces (Qamp versus time) and the averaged modelled single-spike kinetics are represented in the right traces. (**A**), typical time-course secretory curve and a representation of a spike with foot and the parameters measured in each individual spike. (**B**), bulk secretion and spike kinetics in WT and R6/1 CCs [28]; (**C**), bulk secretion and spike kinetics in SOD1^G93A^ CCs [29]; (**D**), cumulative bulk secretion was not monitored in this study; overlapping WT and APP/PS1 spikes are represented on the right part of this panel [33]. See text for further explanation.

Peripheral sympathetic activity has been shown to have been altered in some NDDs, through the monitoring of the circulating levels of noradrenaline and adrenaline; however, scarce and controversial data have not drawn a clear picture. Clearer are the data obtained in adrenal medullary chromaffin cells that in various studies exhibited alterations of bulk exocytosis of catecholamines and distorted kinetics of exocytotic single-events amperometrically monitored, as summarized in Figure 2. So far, these studies have been performed in CCs from three animal models of NDDs, namely APP/PS1 (AD), SOD1^G93A^ (ALS), and R6/1 (HD). Furthermore, proteins associated with PD (α-synuclein) or HD (HAP1) expressed in CCs have also been shown to perturb the kinetics of exocytosis. This cumulating evidence supports the hypothesis that NDDs are not only affecting specific collections of neurons at the CNS: peripheral neuron-like CCs of the adrenal gland are also deeply affected. As CCs are extremely sensitive to changes in blood pH, pO2, Ca^2+^, K^+^, hormones, metabolic factors, neurotransmitters–and because these parameters may be modified in NDDs–altered excitability, ion currents, [Ca^2+^]_c_ signaling, and exocytosis are expected to occur both in resting conditions and during stress. These potential changes are food for thought for future experiments in the many mouse models of NDDs available.
